# MicroRNAs—Are They Possible Markers of Allergic Diseases and Efficient Immunotherapy?

**DOI:** 10.3390/ijms27020902

**Published:** 2026-01-16

**Authors:** Krzysztof Specjalski, Marek Niedoszytko

**Affiliations:** Department of Allergology, Medical University of Gdańsk, 80-210 Gdańsk, Poland; mnied@gumed.edu.pl

**Keywords:** miRNA, allergy, immunotherapy, biomarkers, asthma, allergic rhinitis, atopic dermatitis

## Abstract

Micro-RNAs (miRNAs) are short, non-coding RNA molecules regulating genes’ expression. Studies published over last years demonstrated that they play an important role in allergic diseases by regulating humoral and cellular immunity, cytokine secretion and epithelium function. Some of them seem potential non-invasive biomarkers facilitating diagnosis of the most common allergic diseases, such as allergic rhinitis (miR-21, miR-126, miR-142-3p, miR-181a, miR-221), asthma (miR-16, miR-21, miR-126, miR-146a, miR-148a, miR-221, miR-223) and atopic dermatitis (miR-24, miR-124, miR-155, miR-191, miR-223, miR-483-5p), or objectively assessing severity of inflammation and endotype of the disease. In spite of the large body of literature available, its scientific value is limited due to the small numbers of study participants, heterogeneity of populations enrolled, and diverse methodology. Some studies have revealed significant changes in miRNAs’ profile in the course of allergen immunotherapy. Tolerance induction is associated with processes controlled by miRNAs: enhanced activity of Treg cells and increased production of tolerogenic IL-10 and TGF-β. Thus, miRNAs may be candidates as biomarkers of successful immunotherapy. Finally, they are also possible therapeutic agents or targets of therapies based on antagomirs blocking their activity. However, so far no studies are available that demonstrate efficacy in overcoming delivery barriers, tissue targeting or drugs’ safety. As a consequence, despite promising results of in vitro and animal model studies, translation into human therapeutic agents is uncertain.

## 1. Introduction

MicroRNAs (miRNAs) are short, non-coding RNA chains that are 18–22 nucleotides long and feature a sequence that has been very conserved throughout evolution [1]. Their function remained uncertain until the 1990s when it was discovered that one of them, lin-4, is able to block expression of the genes in *Caenorhabditis elegans* by interaction with another RNA molecule [2]. Currently miRNAs are demonstrated to constitute an effective system of regulating genes’ expression.

Initially, miRNAs are transcribed in a nucleus as long primary miRNA (pri-miRNA). In the next step the transcripts are cleaved by enzyme Drosha to liberate precursor miRNA (pre-miRNA). Precursors are exported to cytoplasm by exportin-5 in the form of a hairpin, which is subsequently processed by enzyme Dicer. The loop sequence from the hairpin is removed. The double-stranded RNA is passed to Argonaute protein where one strand is discarded. Mature miRNAs can exert their activity in the cells where they have been produced or be secreted in extracellular vesicles. The latter is a form of extracellular communication leading to propagation of signals between cells [3].

By binding to the region of 3′ messenger RNA, they downregulate gene expression by promoting degradation of mRNA or inhibiting the process of translation. The first and most obvious consequence is gene silencing, resulting in lower production of protein, e.g., a cytokine. Targeting positive regulators inhibits a process (negative feedback, e.g., blocking IL-5 secretion limits eosinophilic inflammation). In other situations, miRNAs inhibit negative regulators enhancing some processes (positive feedback, e.g., miR-155 inhibits SOCS1 to enhance cytokine signalling). MiRNAs’ activity regulates numerous phenomena, mainly proliferation and differentiation of cells, their pro- or anti-inflammatory activity, intracellular signal transduction, stress response and apoptosis. Relations between miRNAs and gene expression are usually complex and require cautious interpretation as a single miRNA molecule targets identical sequences located in distinct genes and vice versa—a single gene may be regulated by multiple miRNAs [4].

Since the beginning of the current century, thousands of studies have been conducted aiming at investigating the role of miRNAs, particularly in oncogenesis and inflammatory diseases, including allergies and asthma. In several in vitro and animal studies, numerous miRNAs have been shown to affect Th1/Th2 balance, regulate inflammation and remodelling of tissues and activate innate immune cells [5]. Human data demonstrate that some of them correlate with the presence or severity of various diseases and are considered potential biomarkers facilitating diagnosis or prognosis of clinical course [6,7,8]. On the contrary, it is also well known that several miRNAs demonstrate anti-inflammatory and tolerogenic activity [9]. As a result they have been investigated as treatment agents or adjuvants in immunotherapy [10,11].

In this review, the role of miRNAs in T2-high allergic diseases will be discussed with particular emphasis on the possibility of practical clinical application in diagnosing allergic diseases and monitoring therapy.

## 2. Expression of miRNAs in Common Allergic Diseases

### 2.1. Allergic Rhinitis

Allergic rhinitis (AR) is defined as chronic inflammation of nasal mucous membranes initiated by IgE-dependent reactions to allergens. The disease is characterized by eosinophilic infiltrations and overexpression of Th2 cytokines, including IL-4 (promoting T2-type cellular responses and activating IgE production), IL-5 (promoting differentiation and maturation of eosinophils and prolonging their lifespan) and IL-13 (enhancing mucus secretion and secretion of IgE). Some cytokines secreted by macrophages and monocytes (IL-1, IL-6, tumour necrosis factor α (TNFα)) are not specifically related to allergic responses, but have been demonstrated to support and prolong inflammation. On the other hand, IL-10 and transforming growing factor β (TGFβ) both have pro-tolerogenic and anti-inflammatory activity [12,13].

**Figure 1:**
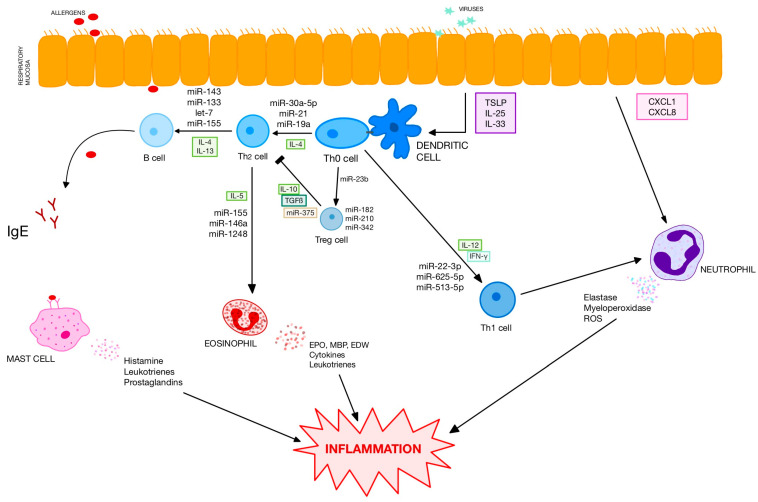
Influence of selected miRNAs on the most important pathways of allergic inflammation.

In patients with AR profiles of miRNA expression in nasal biopsies, extracellular vesicles and serum differ significantly from healthy controls [14,15,16]. The authors have demonstrated dozens of differently expressed miRNAs including downregulated let-7 family and miR-224 and upregulated miR-498, miR-155, miR-223 and miR-205. Differently expressed miRNAs have numerous targets (Figure 1). Several studies have shown that some processes and pathways are particularly influenced by differently expressed miRNAs (B-cell and T-cell receptor signalling, activity of natural killer cells, migration and chemotaxis of leucocytes, formation of extracellular vesicles, etc.) [16]. As miRNAs exert well-known regulatory effects on cytokine secretion, their dysregulation in AR is associated with T1/T2 imbalance in serum with increased secretion of IL-4 and IL-5 and suppression of T1-related cytokines (Table 1).

Interestingly, in patients with allergic rhinitis, miRNA expression is significantly changed. It is well known that transfer of cytokines or chemokines by means of exosomes to adjacent cells significantly modifies their function, e.g., by spreading or silencing of inflammation. It seems that miRNAs can also use exosomes as carriers, spreading their regulatory effect in the neighbouring cells or other tissues [16].

The studies cited above vary in terms of material tested (nasal biopsies, blood, serum) and detection methodology. It is particularly important that study samples are usually small and, as a result, more prone to bias. Thus, some results should be interpreted cautiously, as preliminary data. However, in cases of some miRNAs, results are repetitive and reliable. These molecules have been demonstrated to be potential biomarkers of AR. Among several candidates, the role of miR-181a seems the best documented. First, levels of miR-181a are significantly lower in sera of children with AR compared to healthy individuals (*p* < 0.001). Second, they correlate negatively with some clinical features of disease, such as severity of symptoms measured by TNSS (r = −0.66; *p* < 0.05). Finally, relations have been found between miR-181a and well-known regulators of inflammation. There have been negative correlations with osteopontin (r = −0.58 *p* < 0.05) and Th2 cytokines IL-4 (r = −0.65; *p* = 0.01), IL-5 (r = −0.73; *p* = 0.03) and positive correlations with Th1 cytokines IL-12 (r = 0.754; *p* = 0.04) and IFNγ (r = 0.623; *p* = 0.02) [29]. Most of these relations have been confirmed in distinct allergic populations and seem to not be affected by age, season or medications used [30]. What is more, several studies have aimed at determining a reliable, predictive model based on a small group of distinct miRNAs that would confirm diagnosis of AR and correlate with disease severity. One study has found that a combined assessment of three miRNAs (miR-126-5p, miR-19a-5p and miR-26a-5p) makes it possible to establish AR diagnosis with a high area under the curve (AUC) of 0.866 (95% CI: 0.797–0.936), sensitivity of 90% and specificity of 70%. This subset also correlates with severity of AR [6]. In another study, seven candidate miRNAs selected on the basis of earlier studies have been assessed in a group of 85 patients with confirmed AR and in 57 non-atopic healthy controls [7]. Expression of miR-221 and miR-142-3p was upregulated in the AR group. Their combined evaluation has been characterized by good performance in distinguishing between AR and healthy groups (sensitivity—81%; specificity—65%).

In some studies, authors attempted to apply miRNA evaluation to long-term prognosis. For example, a large panel of 157 miRNAs was evaluated in leucocytes from human umbilical cord blood characterized by increased levels of total IgE. The data were confronted with development of allergic diseases in subsequent years. The authors found the expression of miR-21 and miR-126 to be downregulated in monocytes of patients who were later diagnosed with AR [31].

### 2.2. Asthma

Asthma is a chronic inflammatory disease of the lower airways, leading to recurrent episodes of breathlessness, cough, wheezing and chest tightness as well as, in the longer term, remodelling of the airways. Patients with asthma are a heterogenous group with diverse patterns of inflammation (eosinophilic, which is the most common; neutrophilic; mixed type or paucigranulocytic), treatment responsiveness and concomitant diseases affecting their course of asthma (obesity, smoking, GERD, NERD) [32].

The general asthmatic population is characterized by overexpression of several miRNAs in serum, including miR-16, miR-21, miR-125b, miR-126, miR-145, miR-146a, miR-148a, miR-221, miR-223, miR-338, and miR-485-3p [33,34,35,36]. In nasal biopsies from patients with asthma, researchers have found downregulation of miR-18a, miR-126, let-7e, miR-155, and miR-224 and upregulation of miR-498, miR-187, miR-874, miR-143, and miR-886-3p [15].

MiRNAs play a significant role in asthma on several levels (Table 2). First of all, they modulate chronic inflammation by regulating secretion of cytokines and proinflammatory function of cells (eosinophils, neutrophils). Eosinophils are key inflammatory cells in the T2-high asthma endotype. Their proliferation and survival are controlled by miR-21 and miR-223 [37,38]. In animal models, progenitors of eosinophils lacking miR-21 are characterized by an increased apoptosis rate during maturation. Mice with silenced miR-21 have reduced blood eosinophil count. On the contrary, miR-223-deficient animal eosinophil progenitors are found to proliferate faster.

Neutrophilic asthma is characterized by overexpression of miR-199a-5p, miR-223-3p, miR-142-3p and miR-629-3p in induced sputum [53,54]. MiR-629-3p is expressed in the airways, in epithelial cells. It has proinflammatory properties as it increases secretion of IL-8, a cytokine promoting activity of neutrophils. MiR-223-3p and miR-142-3p have been found in neutrophils, monocytes and macrophages. Their exact role in neutrophilic inflammation is uncertain. Expression of miR-199a-5p is found to correlate with the presence of pulmonary obstruction [53]. Another miRNA controlling the function of neutrophils is miR-3164, altering levels of myeloperoxidase and elastase as well as the process of NET formation [52].

Secondly, the function of bronchial epithelium in asthma is affected by miRNAs. Patients with untreated asthma have different expression patterns of 217 miRNA when compared to healthy individuals. The differences include members of the miR-34/449 family (miR-34b-5p and miR-34c-5p) repressed by IL-13 [55]. Asthmatic patients show upregulation of miR-19a and downregulation of miR-181-5p in bronchial epithelium. The former is related to cell proliferation through targeting TGFβR2 mRNA [56]. MiR-181-5p inversely correlates with both sputum and bronchial submucosal eosinophilia. It targets the osteopontin gene, what affects expression of several cytokines and chemokines (IL-13, IL-1β, CCL11) [42].

Finally, miRNAs contribute to chronic asthmatic inflammation and airway wall remodelling by enhancing hypertrophy and hyperplasia of smooth muscle cells (SMC) [43]. Alexandrova et al. have demonstrated downregulation of 31 and upregulation of 9 miRNAs in asthmatic SMC. Among 363 mRNAs targeted, there have been several transcripts associated with inflammatory and remodelling responses, sensitization to allergens and bronchoconstriction [57]. The best documented molecule regulating SMC function is miR-145 upregulated in T2-high inflammation. Its blocking with the use of antimir resulted in decreased proliferation and migration of SMC, as well as decreased synthesis of collagen I and myosin heavy chain (MHC). Inhibition of miR-145 increases levels of Kruppel-like transcription factor 4 (KLF4) and decreases matrix metalloproteinases-2 and 9 (MMP-2, MMP-9) [58].

Several attempts have been made to determine the miRNA candidates for asthma biomarkers in blood, sputum, exhaled breath condensate, airway extracellular vesicles and saliva [59,60,61]. Milger et al. assessed performance of miRNA selected on the basis of previously known murine models of asthma. No single miRNA made it possible to distinguish with satisfactory sensitivity and specificity between healthy subjects and patients with asthma. However, the combination of five miRNA ratios (miR-21-5p/miR-15a-5p; miR-27a-3p/miR-15a-5p; miR-29c-3p/miR15a-5p; miR-223-3p/miR-425-5p; miR-15a-5p/miR342-3p) seemed a reliable asthma biomarker with AUC of 0.92 [62]. In another study, 30 miRNAs have been found to be expressed differently between patients with asthma and AR and healthy controls (*p* < 0.05). Specific for asthma was upregulation of miR-16, miR-223, miR-148a and miR-146a, and downregulation of miR-299-5p, miR-570 and miR-150. The model based on the six most relevant miRNAs (miR-125b, miR-16, miR-299-5p, miR-126, mir-206, miR-133b) allowed differentiation of healthy subjects and those with asthma and allergic rhinitis with a sensitivity of 92.4% (AUC = 0.936) [34]. Upregulation of miR-223-3p, miR-191-5p and miR-197-3p differentiated patients with asthma and healthy controls with AUC of 0.813; sensitivity of 76%; and specificity of 72% [63]. Applications of miRNA-based models have also been studied in long-term prognosis of treatment outcomes. The model based on 12 variables (including miR-146b-5p, miR-106a, miR-126, miR-30a) applied in 160 children with asthma aged 5–12 years allowed prediction of asthma remission by the age of 14 with sensitivity of 84%, specificity of 70%, and AUC of 80% [8]. A model of five miRNAs (miR-7-5p, miR-155-5p, miR-320b, miR-342-3p, miR-484) correlated with good response to benralizumab in severe asthma cases with AUC for miR-7-5p of 0.905 [64].

The profile of miRNA in exhaled breath condensate allows discrimination between asthma and COPD [61]. Analysis of extracellular vesicles has proven differentiation between asthmatic and health children [60]. In another study, such an approach has made it possible to distinguish between asthmatic and healthy groups and severe and non-severe asthma, as well as find correlations between several miRNAs’ expression and clinical features such as exacerbation rate, asthma control test, and airway obstruction [59].

Some miRNAs have been found to correlate with clinical features of asthma and well-established biomarkers. For instance, miR-296-5p, miR-16-5p, miR-203 and miR-30d-5p correlate with presence of bronchial hyperresponsiveness. MiR-155 correlates with total serum IgE, fractional exhaled nitric oxide (FeNO), and nasal nitric oxide (NNO) [65]. In animal models of asthma, the authors have found relations between the expression of three miRNAs (miR-155, miR-21 and miR-18a) and concentrations of Th2 cytokines in bronchoalveolar lavage [66].

### 2.3. Atopic Dermatitis

Atopic dermatitis (AD) is a common, chronic inflammatory disease of the skin characterized by dryness, pruritus and a typical appearance and distribution of a rash following a relapsing and remitting course. It is widely believed that development of AD stems from the mutual interactions between several genetical and environmental factors. Genetical studies have identified a key role of genes associated with skin barrier function. The best documented ones are filaggrin gene mutations, leading to loss of its function. Their presence strongly correlates with early beginning of AD, more severe skin eruptions and higher incidence of allergic comorbidities [67]. On the other hand, predominance of T2-high responses is an important phenomenon and nowadays it is successfully targeted by biologicals.

As atopic dermatitis shares inflammatory pathways with other T2-high allergic diseases, most of the studies confirm that in both skin and serum of patients with AD there are several upregulated miRNAs known for their involvement in asthma or allergic rhinitis (e.g., miR-21, miR-146a, miR-203) [16]. MiR-483-5p is upregulated in the sera of children with AD, but only in cases with other concomitant atopic diseases [17]. Several studies have demonstrated dysregulation of serum miRNAs associated with T2 cytokines (miR-143) or IL-6 (miR-451a) or activation of inflammation through the NF-KB pathway (miR-146a, miR-124) [68,69]. Other miRNAs, such as miR-720, are upregulated solely in AD, possibly due to organ-specific distribution and functions [70]. miR-10a-5p and miR-29b control keratinocyte proliferation and play a role in skin barrier function [69].

The best documented miRNA related to AD is miR-155 upregulated in skin lesions and patients’ sera [71]. MiR-155 targets cytotoxic T lymphocyte-associated protein 4 (CTLA-4), which is a major inhibitory molecule of T-cell responses. Blockade of CTLA-4 promotes allergic responses with eosinophilia and elevated IgE levels, while increased CTLA-4 inhibits allergic inflammation [72,73]. The receiver operating characteristic curve analysis for miR-155 demonstrates that it can differentiate between children with mild AD and those with moderate-to-severe AD, with an area under the curve of 0.879. A statistically strong significant positive correlation existed between miR-155 levels and SCORAD severity index (rs = 0.666, *p* < 0.001) [74].

The candidates for possible AD biomarkers include miR-24 and miR-191 that are also related to severity of the disease. In the study of Maeno, serum expression of selected miRNAs has been compared between patients with atopic dermatitis (n = 21), controls with chronic urticaria (n = 10) and healthy individuals (n = 13). Patients with severe AD (evaluated with EASI scale) were characterized by higher expression of miR-24 and miR-191 compared to cases with mild AD and control groups. Importantly, differences were insignificant after efficient topical therapy [75]. miR-146a correlates with total serum IgE in patients with AD (r = 0.25; *p* < 0.05) [76]. Interestingly, early development of AD is associated with overexpression of miR-146b, miR-21, miR-22 and miR-375 in mothers’ breast milk [77]. Role of the most important miRNAs in the course of AD is summarized in Table 3.

### 2.4. Other Allergic Diseases

The role of microRNA in the development of less common allergic diseases has not been well investigated.

Patients with allergic conjunctivitis are characterized by lower expression of miR-223. The molecule has been found to negatively correlate with proinflammatory cytokines and serum IgE [78]. In a mouse model of allergic conjunctivitis, miR-146a has been demonstrated to be suppressed [79]. MiR-146a inhibits inflammatory responses, decreases expression of IL-5 and IL-13 and reduces clinical symptoms of conjunctivitis [20]. Although the number of studies on allergic conjunctivitis is limited, their results are mostly in line with data on allergic rhinitis. Both diseases share the T2-related mechanism of inflammation and coexist in a large portion of patients affected.

In acute IgE-mediated reactions, the profile of miRNAs changes temporarily and normalizes with its resolution [80]. In the study of Borque et al., 21 differently expressed miRNAs have been found between anaphylaxis and baseline serum samples. Interestingly, decreased miR-375-3p reversely correlated with two proinflammatory cytokines: GM-CSF and monocyte chemoattractant protein (MCP-1). MiR-375-3p is related to endothelial barrier maintenance [81]. In children with food-induced anaphylaxis, overexpression of miR-21-3p and miR-487b-3p has been demonstrated in extracellular vesicles isolated from acute-phase blood samples [82].

Eosinophilic esophagitis (EoE) is a chronic inflammatory esophageal disorder with eosinophilic infiltrations and non-IgE-mediated food hypersensitivity. Dysregulation of miRNAs associated with T2-high inflammation, such as miR-155, has been documented [83]. Changes in miRNA profile in serum and tissue biopsies are significant in uncontrolled inflammation and subside after introduction of therapy with proton pump inhibitors or steroids [84,85]. Several studies have aimed at finding complementary, non-invasive EoE biomarkers. A lack of reliable biomarker results on the dependency on endoscopy with biopsies for both diagnosis and monitoring. MiR-21-5p and miR-223-3p in plasma and tissue biopsies have been shown to effectively differentiate patients with EoE and controls with GERD (*p* < 0.005) and correlate with successful treatment [84]. In another study, miR-30a-3p has been demonstrated as the best marker for monitoring the course of EoE, and combined assessment of miR-30a-3p and mioR-15a-5p has shown the best sensitivity and specificity on diagnosis; AUC = 0.71 95%, CI 0.58–0.83 [86].

Little is known about the regulatory role of miRNAs in contact dermatitis. Yuehui et al. have shown changing serum expression of let-7d-5p, miR-24-3p, miR-23b-3p, miR-26b-5p and miR-150-5p in female patients with allergy to metals undergoing patch tests [87]. In another study it has been demonstrated that miRNA profile in human skin is significantly influenced by exposure to allergens, with no response upon irritants inducing skin eruptions [88].

## 3. Role of miRNAs in Allergen Immunotherapy

### 3.1. Immunotherapy in IgE-Mediated Diseases

Allergen immunotherapy is a causative method of treatment widely used in IgE-mediated diseases. Immunologic tolerance is induced by administration of increasing doses of allergen (initial phase) and, consecutively, the effect is sustained by administration of stable, high doses over 3–5 years (maintenance phase). Immunotherapy modulates both humoral and cellular responses leading to restoration of allergens’ tolerance. The process is long-term and gradually influences several elements of the immune system. The earliest results of therapy are mostly related to desensitization of effector cells (mast cells and basophils). Intermediate effects are associated with changing activity of allergen-specific T cells. Finally, late effects of immunotherapy involve reduction in IgE secretion as well as an altered function of mast cells, eosinophils and basophils [89]. However currently it is widely believed that the most important in the process of tolerance induction is activity of tolerogenic T regulatory cells suppressing Th2 cells and secreting IL-10 and TGFβ. These cytokines suppress effector cells and change the profile of humoral response from IgE- to IgG_4_-dominated, which does not elucidate an allergic reaction [90].

Allergen immunotherapy has been demonstrated highly effective in allergic rhinitis, asthma, allergy to insect venom and some phenotypes of atopic dermatitis [91,92]. It leads to significant reduction of symptoms and amelioration of disease-related quality of life. The clinical effect is long-term and has been proven to be significant even years after discontinuation [93]. In cases of insect venom allergy, immunotherapy is highly efficient in prevention of stinging-related anaphylaxis [94]. Thus, it can be considered a life-saving procedure. What is more, immunotherapy modifies the natural history of an allergic disease by preventing new sensitizations and reducing the risk of asthma development in patients with allergic rhinitis [95,96]. Up until now, the response to allergen immunotherapy could not be routinely assessed by any objective method, reflecting the complex mechanism of tolerance induction. In everyday clinical practice, the response is evaluated by symptom scores and the treatment required. For scientific purposes, including clinical trials, challenge tests may also be applied. Although they are reliable, it is not possible to use them in everyday practice due to safety concerns (they involve exposure to allergens) and significant time burden.

### 3.2. Expression of miRNAs During Allergen Immunotherapy

Induction of allergen tolerance is a long-term complex process modulating functioning of both humoral and cellular responses. As a consequence it leads to changes in expression of multiple miRNAs. In the study of Specjalski et al., full blood expression of 740 miRNAs was assessed twice: before starting wasp venom immunotherapy (VIT) and 24 h after completing its initial phase (ultra-rush). Statistically significant changes were found in the expression of five miRNAs (miR-370, miR-539, miR-502-3p, miR-299 and miR-29c). Another 62 miRNAs changed two-fold in the majority of patients, including increases in miR-143 and let-7d and decreases in proinflammatory miR-301, miR-146b, miR-106, and miR-485 [97]. In the next step the authors determined the expression of 96 selected miRNAs at three time points: before immunotherapy, 24 h after ultra-rush phase and after 3 months of maintenance phase. The authors found changing expression of 17 out of 96 miRNAs tested, including 8 miRNAs changing their expression as early as 24 h after completing the initial phase [98]. This is in line with some studies demonstrating decreased levels of IL-4 with an increase in the number of IL-10^+^ T cells and higher proportion of CD4 + CD25^+high^ regulatory cells just 3.5 h after ultra-rush [99]. Importantly, all early changes in miRNA expression were also confirmed three months later. The changes that could have a significant impact on immunotherapy included upregulation of let-7d (member of anti-inflammatory and tolerogenic let-7 family), miR-143 (increasing FOXp3 expression and repressing IL-13), and miR-34b (downregulated by IL-13 in AR and asthma) and downregulation of miR-375 (regulating production of IL-13), miR-342 (regulating NFκβ expression and function of Treg cells), and miR-182 (regulator of Th17 and Treg cell function) [98]. However, the clinical relevance of changes in miRNA profile is uncertain. The participants were not sting-challenged, nor did they experience field stinging. As a result, successful tolerance induction was not confirmed and could only by assumed by the fact that in the vast majority of patients VIT was efficient from the completion of its initial phase.

The profile of miRNAs was also investigated during allergen immunotherapy with airborne allergens. In patients with perennial AR, immunotherapy with house dust mite extracts led to suppression of miR-19a. It has been well documented that miR-19a is upregulated in AR. Its secretion is increased by IL-4 in a dose-dependent manner and negatively regulated by IL-10 [100]. In another study, immunotherapy with grass pollen was associated with changing expression of several miRNAs in induced sputum. Among others, miR-3935 was upregulated. The target for this molecule is prostaglandin EP3 receptor [101].

In the study of Luo et al., immunotherapy was introduced due to allergic rhinitis related to house dust mite allergy. Herein, 24 children were allocated either to sublingual (SLIT) or subcutaneous immunotherapy (SCIT). After 3 months of therapy an increased expression of miR-146a was revealed in both groups. In patients’ sera, there was significant increases in IL-10 and decreases in IL-5 levels irrespective of the immunotherapy method applied. However, the authors have not found any correlation between changing expression of miRNAs and a clinical response to the immunotherapy [11]. Another study assessed miRNA expression in patients with allergy to Japanese cedar pollen undergoing SLIT. Out of 15 participants, 6 underwent sublingual immunotherapy with the cedar pollens and 9 received placebo only. The therapy was successful in the actively treated group. During a pollinating season, seven subjects in the placebo group and none administered SLIT developed symptoms of rhinitis, respectively. MiRNA levels in patients’ sera were determined in pre- and postseasonally. The only miRNA that changed significantly was miR-223, with no differences between the SLIT and placebo groups. In the placebo group development of pollinosis was significantly associated with downregulation of let-7b [102]. Although placebo-controlled, this study is underpowered (small sample, short follow-up). Moreover, limited findings may have stemmed from the fact that the authors relied only on serum assessment, related rather to systemic responses, instead of investigating local miRNA expression in nasal mucosa.

A Polish study investigated expression of 48 selected miRNAs in sera of patients with AR undergoing SCIT with grass pollen, patients with AR treated symptomatically and healthy controls. Hierarchical variance analysis showed 27 changes related to SCIT. Most of them were observed in miRNAs controlling Th1/Th2 balance or immunologic tolerance. After 6 months of study participation, the group undergoing SCIT did not differ from healthy controls any longer—expression of miR-208, miR-190 and miR-136 that previously had been upregulated was comparable. However some new differences appeared between the group on SCIT and the group treated symptomatically in terms of miR-483-5p and miR-136, which were downregulated in the SCIT group. MiRNA expression was evaluated outside of the pollinating season in order to avoid effects of acute allergen exposure. The study has not revealed any difference in miRNA expression between good and poor responders who differed significantly in terms of symptoms and medication scores. Thus, despite significant changes in expression, miRNAs’ utility as response biomarkers has not been proven [103].

Finally, Zhang et al. investigated miRNA profile in serum exosomes in patients undergoing SLIT due to AR. They found that miR-146a-3p was upregulated in patients with allergic rhinitis. It correlated with total IgE, specific IgE and symptom severity. Upon SLIT receipt, this molecule was suppressed, which correlated with clinical response. Thus, authors concluded that miR-146a-3p could be a candidate marker of successful tolerance induction in immunotherapy [104].

Although immunotherapy is related to several changes in the expression of miRNAs, so far few correlations have been found between their expression and clinical outcome. Thus, despite some promising studies in this field, no miRNAs has been found to be a good biomarker indicating good efficacy of allergen immunotherapy.

The major limitation of the studies discussed is the relatively small number of participants enrolled. Authors recruit different groups of patients (seasonal or perennial symptoms, isolated AR or AR with asthma), use distinct methods (SCIT, SLIT) and allergens (pollens, house dust mites) and determine miRNAs in distinct material (blood, serum exosomes). Furthermore, clinical response to immunotherapy is usually delayed and should be assessed no sooner than several months or years after introduction of treatment. To our knowledge no study assessed changing profiles of miRNAs repeatedly over such a long period of time. Another limitation of available studies is analysis of miRNAs solely, without integration with other biomarkers, either well-established or candidates such as mRNA. In predicting clinical outcomes in oncology, approaches based on single-cell transcriptomics or bulk transcriptomics have proven very effective. They enabled dividing patients into several immune subtypes with varied response to therapy [105,106]. Such a strategy seems possible in allergen immunotherapy as well. Whole-genome expression profiling in peripheral blood mononuclear cells has demonstrated that immunotherapy induces rapid and dynamic alteration in both innate and adaptive immunity [107]. In both SCIT and SLIT there is a significant change in expression of genes related to T1/T2 balance, particularly IL-4, IL-5, IL-10, TGFβ, TNF, and IFNγ [108]. Some of the transcripts are potential candidates for biomarkers of successful immunotherapy [109].

Finally, in this review we attempted to present available evidence of the utility of miRNA application despite the fact that the methodology of the studies cited has some faults. To improve scientific robustness it would be recommended to implement harmonized protocols regarding biobanking, QC metrics and statistical pipelines, assuring transparent and reproducible data.

## 4. Perspectives for the Future Application of miRNAs in Allergic Diseases

Despite the significant progress in fields of biology, immunology and genetics, making diagnosis of allergic diseases is still largely based on traditional, clinical criteria. For example, atopic dermatitis can be confirmed with the use of Hanifin and Rajka criteria. According to the GINA report, asthma should be diagnosed on the basis of clinical characteristics and proof of reversible airway obstruction [32]. As a matter of fact no biomarkers are routinely used in diagnosing common allergic diseases. As a result, diagnosis is often based on subjective assessment of symptoms, or cannot be confirmed at all (e.g., pulmonary function tests are not possible in babies). Another unmet need is objective long-term monitoring that would reflect severity of inflammation and response to treatment. MicroRNAs controlling various pathways of allergic inflammation have potential to address these issues. In previous sections several promising candidates for biomarkers of asthma, allergic rhinitis and atopic dermatitis have been presented. However, still more studies are needed that aim at finding correlations between miRNA profile, immunologic changes and clinical outcomes. Considering the fact that allergic diseases are chronic and over the years patients experience remissions and relapses, exacerbations and stabilizations, we need longitudinal, multi-centre studies assessing performance of miRNA biomarkers in real-life settings, in substantial groups of patients. What is more, so far microRNA expression has usually been tested solely. Potential integration of miRNA with RNA markers or a multi-omics strategy is a promising alternative that could better predict response to therapy and the clinical course of the disease.

As miRNAs control the functioning of all types of immunity, it would be tempting to use them as therapeutic agents in order to modify cytokine secretion or proinflammatory activity of effector cells, among others. So far miRNAs have mostly been assessed in in vitro studies or in animal models. The possible interventions included application of miRNAs or agomirs (i.e., synthetic, chemically modified RNA molecules mimicking a natural miRNA). Another option is using antagomirs, which are chemically engineered, antisense oligonucleotides complementary to the specific miRNA target. Having bound miRNAs, they lead to their functional inhibition and derepression of mRNA targets.

In in vitro studies, miR-143, which is downregulated in patients with AR, suppressed expression of genes coding IL-13 receptor α1 chain (IL-13Rα1) in nasal mucosa cells, whereas miR-106b supressed pro-allergic activity of dendritic cells [110,111,112]. MiR30a-5p targeted suppressor of cytokine signalling 3 (SOCS3), necessary for the process of T helper cell differentiation and involved in the pathomechanism of allergic rhinitis [113]. In vitro application of miR-375 agomir inhibited the JAK2/STAT3 pathway and, as a result, changed cytokines’ profile in nasal mucosa cells (downregulation of IL-6 and TNFα; upregulation of IL-10) [114].

In animals, miRNA administration was investigated mostly using models of AR. MiR-135a, known to downregulate GATA-3 and regulate mast cell degranulation, suppressed infiltrations with eosinophils and mast cells in nasal mucosa of ovalbumin (OVA)-sensitized mice [9]. In another study, miR-135a suppressed IL-4 secretion in nasal mucosa [115]. Intranasal application of miR-133b agomir reduced symptoms of rhinitis in mice, decreased serum levels of total IgE and proinflammatory cytokines (IL-4, IL-5, TNFα) and attenuated eosinophil and mast cell infiltration in nasal mucosa [116].

Interestingly, miRNA administration may modify the process of tolerance induction. Liu et al. attempted to assess the role of miR-146a in modulating immunotherapy in a murine model of rhinitis. Before this study miR-146a had already been shown to be suppressed in both humans and mice sensitized to ovalbumin (OVA). Sensitized mice with symptomatic AR were randomly allocated to intranasal immunotherapy with nanovaccine containing OVA or miR-146a or both. It was demonstrated that nanovaccine containing ovalbumin only led to exacerbation of rhinitis. In contrast, miR-146a had no impact on symptoms. However, when administered together with ovalbumin, it significantly enforced the effect of immunotherapy and led to a reduction of AR symptoms and increased presence of Treg cells in nasal mucosa. The positive effect of therapy correlated with the induction of TGFβ. Its role in immunotherapy is well documented as it induces IL-10, suppressing allergic inflammation in nasal mucosa [10].

There are several potential candidates for adjuvants that could enhance effects of immunotherapy. For example, in vitro miR-23b facilitates tolerogenic activity of dendritic cells and Treg cells, promoting differentiation of Treg cells and enhancing secretion of IL-10 [117]. On the other hand a similar effect could be achieved by blocking proinflammatory miRNAs known as suppressors of Treg cells or promoters of eosinophilic inflammation. The efficacy of antagomirs has been demonstrated in relation to miR-223, miR-126 or miR-21 [38].

Despite the promising conclusions of in vitro studies, impacts of the therapies based on microRNAs or their antagomirs are still quite difficult to predict and interpret. First, activity of miRNAs is usually complex as one molecule has several mRNA targets and, vice versa, a single gene is affected by many miRNAs. As a consequence, administration or blocking of a single miRNA could potentially modify expression of numerous genes with unknown clinical consequences. On the other hand, in multifactorial diseases, even successful silencing of a single gene may not translate into clinical benefits.

Administration of miRNA-based therapeutics requires stable and efficient delivery systems, firstly in order to protect nucleic acid from degradation by nucleases in physiological fluids, and secondly to regulate cellular uptake and miRNA release. Finally, miRNAs must escape lysosomes and reach cytoplasm where translation occurs. Recently, a variety of molecules have been developed and some of them have high potential to be effective RNA carriers. They include lipids, lipid-like materials, polymeric nanoparticles (hydrogels), inorganic nanoparticles and biomimetic nanoparticles (e.g., biomimetic cell membranes) [118].

Out of all particles listed, lipid-based ones seem the most promising so far. They contain cationic lipids with a head group with permanent positive charges (DOTMA, DOTAP, etc.) or neutral, ionizable lipids, often with stabilizing phospholipids, cholesterol or PEG-functionalized lipids. In the last decade a milestone has been reached with the introduction of lipid nanoparticle–mRNA vaccines against COVID-19. Apart from vaccines, such formulations have potential in a range of applications, including protein replacement therapies (e.g., in cystic fibrosis), cancer immunotherapy (e.g., RNA coding OX40L, IL-12, GM-CSF), genome editing or cellular reprogramming [119].

There are still several unanswered questions concerning practical application of therapy based on miRNAs in allergic diseases. First, groups of potential good responders who would benefit mostly from the therapy need to be defined. As most of data presented above refers to the control of T2 inflammation, this group of diseases is probably the first target. Second, there is no defined optimal route of administration of therapies. We do not currently have any comparative studies assessing distinct administration modes. Safety assessments are necessary to determine effective but also specific therapy. Answering these questions requires investigating pharmacokinetics, biodistribution and immunogenicity of molecules tested. Finally, we need longitudinal, placebo-controlled studies assessing long-term response to miRNA-based therapy in terms of symptoms’ control, inflammation silencing, concomitant symptomatic medication used and correlation with already known biomarkers.

## 5. Conclusions

In recent years miRNAs have been demonstrated to regulate allergic inflammation on several levels, such as humoral and cellular immunity, cytokine and chemokine secretion, and the function of epithelium and smooth muscle cells. Some of them seem potential non-invasive biomarkers facilitating diagnosis of allergic rhinitis (miR-21, miR-126, miR-142-3p, miR-181a, miR-221), asthma (miR-16, miR-21, miR-126, miR-146a, miR-148a, miR-221, miR-223) and atopic dermatitis (miR-24, miR-124, miR-155, miR-191, miR-223, miR-483-5p) or objectively assessing the severity of inflammation and endotype of the disease. In the context of allergen immunotherapy, miRNAs are particularly promising candidates. Their profile changes during tolerance induction and may correlate with a reduction in clinical responses to allergen exposure. Finally, they are also a possible target of future therapies based on miRNAs or antagomirs blocking their activity.

Despite the fact that our knowledge about the role of miRNAs has expanded significantly in recent decades, several areas need further investigation. First of all, our knowledge on miRNAs is largely based on cell cultures or animal models. We still do have enough studies focused on human settings. This is particularly important considering the heterogeneity of several human allergic diseases (asthma, AD) and well-described interactions with other pathologies (obesity, GERD, sinusitis, etc.). Another challenge is the possibility of therapeutic application of miRNAs. Apart from the technical issues (stability of miRNAs, vector application, organ-specificity, etc.) there are still unanswered questions concerning the efficiency of such therapy. As relations between miRNAs and genes are quite complex and Th1/Th2 balance is regulated by several miRNAs, it is difficult to predict the clinical effect of a single miRNA applied. Moreover, studies based on animal models cannot be directly translated to clinics.

## Figures and Tables

**Table 1:** Influence of selected miRNAs on immune system in allergic rhinitis.

Target	miRNAs Regulating Expression	Citation
IL-4	miR-155, miR-221-3p	[17,18]
IL-5	miR-155, miR-1248, miR-146a	[17,18,19,20]
IL-13	miR-155, let-7	[17,18,21,22]
T cells (polarization towards Th2)	miR-210, miR-181a, miR-19, miR-21a	[23,24,25,26]
TGFβ	miR-323-3p, miR-181a, miR-26a	[27]
Th1 cytokines (IL-12, INFƔ)	miR-513-5p, miR-22-3p, miR-625-5p	[28]

**Table 2:** Role of selected miRNAs in pathomechanism of asthma.

miRNA	Role in Pathomechanism of Asthma
Bronchial Epithelial Cells	
miR-146a	IgE synthesis; promoting eosinophilic inflammation [39,40]
miR-210	T_reg_ cell function [41]
miR-34a	dendritic cell maturation and function [41]
miR-181	upregulation of proinflammatory cytokines: IL-13, IL-1 [42]
Bronchial Smooth Muscle Cells (SMC)	
miR-10, miR-708, miR-140-3p, miR-142-3p	SMC contractility, hyperplasia, hypertrophy, remodelling [43,44,45]
miR-25	secretion of cytokines and chemokines [46]
miR-133	secretion of IL-13; SMC contractility; bronchial hyperreactivity [47]
Immune System	
miR-210	inhibition of T_reg_ function [23]
miR-181a	augmenting sensitivity of T cells to peptide antigens [25]
miR-21, miR-19a	promoting differentiation of T cells towards Th2 [23,26,48]
miR-221-3p, miR-155	IL-4 upregulation [17,18]
miR-1248, miR-146a, miR-155	IL-5 upregulation [18,19,20]
let-7 family	IL-13 downregulation [21,22]
miR-146a, miR-144-3p, miR-125-5p	Regulation of tolerogenic IL-10 [49,50]
miR-323-3p, miR-181a, miR-26a, miR-31a-5p	TGFß-dependent signalling pathway modulation [27,51]
miR-513-5p, miR-22-3p, miR-625-5p	inhibition of Th1 cytokines including IL-12, and INFƔ [28]
miR-3164, miR-199a-5p, miR-223-3p, miR-142-3p, miR-629-3p	regulation of neutrophilic inflammation [52,53,54]

**Table 3:** Influence of selected miRNAs on pathomechanism of atopic dermatitis.

miRNAs	Role in Atopic Dermatitis	Citation
miR-155	Targets IL-4, IL-5 By inhibition of CTLA-4, controls T-cells responses, IgE production, tissue eosinophilia	[17,18,19,20]
miR-146a	Targets IL-5, promotes tolerogenic IL-10	[17,18,19,20]
miR-451a	Targets IL-6	[68,69]
miR-151a	Targets IL-12R	[28]
miR-143	Targets IL-13R	[68,69]
miR-223	Controls differentiation, function of Treg cells and eosinophils; activation of NF κβ	[69]
miR-720	Controls keratinocyte function and melanogenesis	[70]
miR-10a, miR-29b	Control keratinocyte proliferation and function, epithelial barrier integrity	[69]

## Data Availability

No new data were created or analyzed in this study. Data sharing is not applicable to this article.

## Abbreviations

AD	atopic dermatitis
AR	allergic rhinitis
GERD	gastroesophageal reflux disease
MMP	metalloproteinase
N-ERD	NSAID-exacerbated respiratory disease
OVA	ovalbumin
SCIT	subcutaneous immunotherapy
SLIT	sublingual immunotherapy
SMC	smooth muscle cells
SOCS	suppressor of cytokine signalling
TGF	transforming growth factor
TNF	tumour necrosis factor
